# Earlier Diagnosis of Invasive Fusariosis with *Aspergillus* Serum Galactomannan Testing

**DOI:** 10.1371/journal.pone.0087784

**Published:** 2014-01-28

**Authors:** Marcio Nucci, Fabianne Carlesse, Paola Cappellano, Andrea G. Varon, Adriana Seber, Marcia Garnica, Simone A. Nouér, Arnaldo L. Colombo

**Affiliations:** 1 University Hospital, Universidade Federal do Rio de Janeiro, Rio de Janeiro, Brazil; 2 Institute of Pediatric Oncology (GRAACC), Universidade Federal de São Paulo (UNIFESP), São Paulo, Brazil; 3 University Hospital, Universidade Federal de São Paulo (UNIFESP), São Paulo, Brazil; University of Wisconsin Medical School, United States of America

## Abstract

Cross-reactivity of *Fusarium* species with serum galactomannan antigen (GMI) test has been observed. We sought to evaluate if GMI could help to early diagnose invasive fusariosis and to monitor treatment response. We reviewed the records of all patients with invasive fusariosis between 2008 and 2012 in three Brazilian hospitals. We selected patients who had at least 1 GMI test within 2 days before or after the date of the first clinical manifestation of fusariosis, and analyzed the temporal relationship between the first positive GMI test and the date of the diagnosis of invasive fusariosis, and the kinetics of GMI in relation to patients' response to treatment. We also selected 18 controls to determine the sensitivity and specificity of the test. Among 18 patients, 15 (83%) had at least one positive GMI (median 4, range 1–15). The sensitivity and specificity of was 83% and 67%, respectively. GMI was positive before the diagnosis of invasive fusariosis in 11 of the 15 cases (73%), at a median of 10 days (range 3–39), and after the diagnosis in 4 cases. GMI became negative in 8 of the 15 patients; 3 of these 8 patients (37.5%) were alive 90 days after the diagnosis of fusariosis compared with 2 of 7 (29%) who did not normalize GMI (p = 1.0). GMI is frequently positive in invasive fusariosis, and becomes positive before diagnosis in most patients. These findings may have important implications for the choice of antifungal therapy in settings with high prevalence of invasive fusariosis.

## Introduction

The management of invasive aspergillosis has greatly improved with the introduction of antigen detection [1]. Serum galactomannan antigen (GMI) testing is an important tool for both diagnosis and outcome evaluation of invasive aspergillosis [2]–[5]. Although GMI is considered specific for *Aspergillus* species, cross-reactivity with other fungi, including moulds and dimorphic fungi, has been occasionally reported [6]–[9]. Recently, two papers reported cross-reactivity of GMI with *Fusarium* species. One study presented a case of invasive fusariosis caused by *Fusarium verticillioides* in a hematopoietic cell transplant (HCT) recipient in which serum GMI was positive seven days before the diagnosis of fusariosis [10]. The other study reported 9 out of 11 patients with hematologic diseases and invasive fusariosis with positive GMI tests. The temporal relationship between GMI positivity and the diagnosis of invasive fusariosis was not reported [11].

In the present study we report 18 cases of invasive fusariosis diagnosed in hematologic patients who were actively monitored with serial serum GMI, 15 of which had positive GMI tests. We analyzed the performance of GMI, the temporal relationship between GMI positivity and the diagnosis of fusariosis, and the kinetics of GMI after treatment.

## Patients and Methods

This is a retrospective study conducted in three tertiary care hospitals in Brazil. We reviewed all cases of invasive fusariosis diagnosed in the three institutions since the introduction of active monitoring of high-risk hematologic patients with serial (at least 2 times per week, starting on admission) serum GMI testing. Entry criteria were: to have a diagnosis of proven or probable invasive fusariosis according to the revised EORTC/MSG diagnostic criteria [12], to have at least one GMI test performed within two days before or after the date of the first clinical manifestation attributed to invasive fusariosis, and no isolation of *Aspergillus* species from any biological material. The study was approved by the institutions' ethical committees, and a waiver of the need for written informed consent was granted, because of the observational nature of the study: Comite de Etica em Pesquisa do Hospital Universitário Clementino Fraga Filho and Comite de Etica em Pesquisa da Unifesp/EPM.

Patients were managed according predefined standards of care at each institution. Active monitoring of patients at risk for invasive aspergillosis with serial serum GMI started in 2008, and consisted of GMI testing 2–3 times per week during periods at risk, starting on admission. In case of fever and neutropenia, empiric antibiotic therapy with a beta lactam was started after collection of blood cultures. Additional blood cultures were obtained in case of persistence or recurrence of fever, or at the discretion of the attending clinician. A diagnostic-driven antifungal approach was given to patients with persistent fever, consisting of active monitoring with serial serum GMI and chest and sinuses computed tomography (CT) scans. Serum GMI testing was performed according to the manufacturer's instructions (Platelia Aspergillus EIA; Bio-Rad, Redmond, WA). A positive GMI test was defined by an optical density [OD] index ≥0.5. Blood cultures were processed by automated systems (either Bactec or BacTAlert).

We reviewed patients' charts, results of GMI, blood cultures, histopathology, and results of direct exam and culture of different biological materials that served the basis for the diagnosis of invasive fusariosis. Information from patients included demographics (age, gender), underlying hematologic disease, recent treatment for the underlying disease, presence and duration of neutropenia, receipt of corticosteroids, antifungal prophylaxis, date of the first clinical manifestation attributed to invasive fusariosis, clinical presentation, source of diagnosis, date in which the diagnosis was established, treatment and outcome. We analyzed the temporal relationship between the first positive GMI test and the date of the first clinical manifestation and the date of diagnosis of invasive fusariosis, as well as the kinetics of GMI in relation to patients' response to treatment.

Neutropenia was defined as an absolute neutrophil count (ANC) <500/mm^3^. Disseminated fusariosis was defined as involvement of >1 non-contiguous organs. Cases of fungemia were not defined as disseminated disease unless another organ was involved (e.g. skin, lung or sinuses).

In order to analyze the performance of GMI in the diagnosis of invasive fusariosis, we selected one control for each case and calculated the sensitivity and specificity of the test. Controls had to have received similar underlying diseases and/or treatment as cases, to have had at least two GMI tests performed per week, and no invasive fungal disease diagnosed in the period. The controls were selected blindly for the results of GMI, and a careful review of patients' charts was performed to ascertain that no invasive fungal disease was diagnosed in that period. We also evaluated the best GMI cutoff value constructing the area under the receiver operating characteristic (ROC) curve. Categorical variables were compared using Fisher exact test and continuous variables were compared using the Wilcoxon test.

## Results

Between 2008 and 2012, 25 cases of invasive fusariosis were diagnosed in the three institutions. Seven cases were excluded; four because no GMI tests were performed in the period, and three because the closest GMI test was >2 days from the date of the first clinical manifestation of invasive fusariosis. The main characteristics of the remaining 18 cases are shown in Table 1. Acute myeloid leukemia (AML) was the most frequent underlying condition (6 patients); there were eight HCT recipients (7 allogeneic and 1 autologous). All but one patient was neutropenic.

**Table 1:** Characteristics of 18 patients with invasive fusariosis.

Characteristics	
Gender male: female	16∶2
Age (years), median (range)	46 (10–69)
Underlying disease	
Acute myeloid leukemia	6
Acute lymphoid leukemia	4
Multiple myeloma	2
Other*	6
Recent treatment for the underlying disease	
Chemotherapy	9
Allogeneic HCT	7
Autologous HCT	1
No treatment	1
Neutropenia	17
Receipt of corticosteroids**	7

* Other underlying disease (1 case each): non-Hodgkin's lymphoma, chronic myeloid leukemia, aplastic anemia, myelodysplasia, chronic lymphoid leukemia, and myelofibrosis

HCT =  hematopoietic cell transplantation

** Within 4 weeks before the diagnosis of invasive fusariosis.

Invasive fusariosis was classified as disseminated in 13 patients (72%), fungemia and cutaneous localized infection in two patients each, and arthritis in one patient. Skin involvement was present in 13 patients, pneumonia in 14 (11 of whom with bilateral involvement), sinusitis in four, and arthritis and central nervous system involvement in one patient each. Overall, blood cultures were positive in six patients. Species identification was available in 11 cases: *Fusarium solani* species complex (FSSC) in eight and *Fusarium oxysporum* species complex (FOSC) in three patients.

The diagnosis was made by culture + histopathology in 10 cases, culture alone in seven cases, and histopathology alone in one case. As shown in Table 2, the primary source of diagnosis was skin (10 patients), followed by blood (5 patients). The fastest way of reaching a presumptive diagnosis was by direct exam of a skin biopsy (7 patients) or other biological material (sinus aspirate and bronchoalveolar lavage, one patient each). In these cases, the finding of hyaline hyphae in the direct exam was reached just a few hours after the procedure performed for obtaining the samples, and prompted the immediate initiation of antifungal treatment. The median time to positivity of blood culture was three days (range 1–4). In two cases the diagnosis was established >1 week after the diagnostic procedure; in both the direct exam of a skin biopsy was negative and the diagnosis was established by histopathology or culture + histopathology.

**Table 2:** Source of diagnosis and temporal relationship between the first positive galactomannan and diagnosis in 18 patients with invasive fusariosis.

Patient	Primary source of diagnosis	Direct exam	Culture	Histopathology	Time (days) from procedure to diagnosis	Time (days) from 1^st^ serum GMI to diagnosis
1	Skin	Positive	Positive	Positive	0	−16*
2	Skin	Positive	Positive	Positive	0	+4
3	Skin	Positive	Positive	Positive	0	−1
4	Skin	Positive	Positive	Positive	0	−13
5	Skin	Positive	Positive	Positive	0	−14
6	Skin	Positive	Positive	Positive	0	−8
7	Skin	Positive	Positive	Positive	0	−6
8	Skin	NP	Positive	Positive	8	Negative
9	Skin	Negative	Positive	Positive	5	+8
10	Skin	Negative	Negative	Positive	11	Negative
11	Blood	NA	Positive	NA	1	−25**
12	Blood	NA	Positive	NA	3	−3
13	Blood	NA	Positive	NA	4	−10
14	Blood	NA	Positive	NA	3	+2
15	Blood	NA	Positive	NA	3	+6
16	Sinus aspirate	Positive	Positive	NA	0	Negative
17	Synovial fluid	Negative	Positive	NA	4	−39***
18	Bronchoalveolar lavage	Positive	Positive	Positive (skin)	0	−10

GMI =  galactomannan antigen; NP =  not performed; NA =  not applicable; the signal (+) means that serum galactomannan antigen became positive after the diagnosis of invasive fusariosis, and the signal (−) means that serum galactomannan antigen became positive before the diagnosis of invasive fusariosis

* This patient had 4 positive GMI tests before the first clinical manifestation of fusariosis and 7 positive tests before the diagnosis.

** This patient had 2 positive GMI tests 25 days before the diagnosis of fusariosis, and another positive test on the day of a positive blood culture for *Fusarium*. During this 25-day period the patient received anidulafungin for the treatment of a candidemia, and had 5 negative GMI tests, with values >0.4.

*** This patient had 3 positive GMI tests while neutropenic, with focal ground-glass infiltrates in the left lung. Neutropenia resolved and the patient received posaconazole prophylaxis for the subsequent cycle of chemotherapy. Eleven days after the last positive GMI test he complained of pain and edema in the left knee. The edema increased when he became neutropenic, and a puncture was performed 25 days after the first symptom. During this period 5 GMI tests were negative.

All 18 patients received treatment. The most frequent drug used as primary therapy was voriconazole (8 patients), followed by deoxycholate amphotericin B (4 patients). The remaining six patients received combination therapy (lipid amphotericin B plus voriconazole and deoxycholate amphotericin B plus voriconazole, 3 patients each). The 30-day and 90-day survival was 50% and 33%, respectively.

The median number of GMI tests per patient was 13.5 (range 2–24). Fifteen of the 18 patients (83%) had at least one positive GMI test: all three cases caused by FOSC, seven of 10 caused by FSSC, and three of five of *Fusarium* sp. Among the 15 patients with positive GMI, the median number of positive GMI tests per patient was 4 (range 1–15), and the median value of the first positive and peak GMI was 0.778 (range 0.506–1.915) and 0.969 (0.506–6.382), respectively. One of the three patients with negative GMI had 10 GMI tests performed while on voriconazole prophylaxis, which had been given since 28 days before the diagnosis of fusariosis. The other two patients had seven and 13 negative GMI tests, respectively. They were neutropenic, and had disseminated disease with bilateral lung involvement and sinusitis (1 patient) and metastatic skin lesions (1 patient). We did not find any difference between the 15 patients with positive GMI and the three with negative GMI tests.

We analyzed the timing of GMI positivity related to the first clinical manifestation attributed to fusariosis and to the diagnosis of fusariosis (Table 2). GMI was positive before the first clinical manifestation of fusariosis in eight of the 15 patients with positive GMI (53%), at a median of 8 days (range 1–14), on the same day in one case, and after the first clinical manifestation in the remaining six cases, at a median of 6.5 days (range 2––15). Likewise, GMI was positive before the final diagnosis of invasive fusariosis in 11 of the 15 cases (73%), at a median of 10 days (range 3–39), and after the diagnosis in the remaining four cases (2, 4, 6 and 8 days after). The 11 patients with positive GMI before the diagnosis of invasive fusariosis had a median of 2 positive tests before diagnosis (range 1–7), with five patients presenting ≥4 positive tests before the diagnosis. The patients with positive GMI 39 and 25 days before the diagnosis of fusariosis had 4 and 5 negative tests during this period, respectively. Both were receiving anti-mould active antifungal agents in this period (Table 2).

In eight of the 15 patients with positive GMI the test became negative, at a median of 14 days from the first positive test (range 1–25). Three of these eight patients (37.5%) were alive 90 days after the diagnosis of fusariosis compared with two of seven (29%) who did not normalize GMI (p = 1.0). Similarly, there was no correlation between fast (within 7 days from first positive) GMI normalization and 90-day outcome (1 of 3 alive with fast normalization vs. 4 of 12 who normalized later or did not normalize GMI).

Cases and controls were similar regarding age, gender, underlying disease and its treatment, and presence and duration of neutropenia. The median number of GMI tests performed was higher in cases than in controls (13.5, range 2–24 vs. 6.5, range 3–14, p = 0.003). Six controls (33%) had positive GMI (median of 1 positive test, range 1–3). Considering the cutoff of 0.5, the sensitivity and specificity of GMI was 83% and 67%, respectively. The best cutoff value according to the ROC curve was 0.518, with an area under the curve of 0.738 (95% confidence interval 0.557–0.919), sensitivity of 79% and specificity of 72%.

## Discussion

In this study we observed that GMI is frequently positive in patients with invasive fusariosis (15 of 18, 83%), with >1 positive test per episode (median of 4). We also observed that in most patients the test was positive before the diagnosis (73%) or even the first clinical manifestation attributed to invasive fusariosis. The sensitivity of the test was good, and the best cutoff was close to the 0.5 value defined for invasive aspergillosis. Finally, there was no correlation between GMI kinetics and the outcome.

The GMI test is an ELISA-based test that uses the rat monoclonal antibody EB-A2. The antibody recognizes a galactomannan epitope that contains galactofuranose, a polysaccharide present in various moulds, including Fusarium species [13], [14]. Therefore, cross-reactivity of the GMI test with other fungi may occur, Occasional cases of fusariosis with positive GMI had been reported [15]. In a recent study, 12 clinical isolates of different species of *Fusarium* were tested for the reactivity with GMI. All *Fusarium* isolates produced positive reactions, with GMI values ranging from 1.15 to 4.14. In addition, among 11 patients with hematologic malignancies who developed invasive fusariosis (8 proven and 3 probable, 5 due to *Fusarium proliferatum*, 3 due to *F. verticillioides*, 2 due to *F. oxysporum* and 1 due to FSSC) nine had positive GMI tests, with values ranging from 0.53 to 7.7. No information about the time of occurrence of GMI positivity related to the diagnosis of invasive fusariosis or the kinetics of GMI in treated patients was provided. The authors concluded that GMI may be useful for the diagnosis and assessment of treatment response among patients with invasive fusariosis [11]. The temporal relationship with GMI and the diagnosis of fusariosis was reported recently in an allogeneic HCT recipient. The patient was neutropenic and presented two positive GMI tests; four days after the first positive GMI blood culture became positive for *F. verticillioides*. In vitro this isolate tested positive for GMI, as did two other isolates [10].

Our study differs from these two papers for the highest number of patients, the different species distribution, and because we were able to analyze both the temporal relationship between GMI positivity and the diagnosis of invasive fusariosis, and the kinetics of GMI related to the outcome. We found that the majority of patients with invasive fusariosis presented with positive GMI. This is of great importance in regions where invasive fusariosis is frequent [16]. Among the three patients with negative GMI tests, one was receiving voriconazole for almost one month before the diagnosis of invasive fusariosis. Likewise, the two patients with positive GMI tests 39 and 25 days before the diagnosis were receiving anti-mould antifungal agents. This could have rendered the test negative, by decreasing the sensitivity of GMI [17]–[19].

We also observed that not only GMI was frequently positive but the number of positive tests (median of 4) and GMI values (peak value as high as 6.382) were high. These data indicate that neither the number of positive tests nor the actual GMI value is useful to discriminate between invasive fusariosis and invasive aspergillosis. Instead, other parameters should be taken into account, such as the presence of metastatic skin lesions and positive blood cultures, which are far more common in invasive fusariosis [20].

Invasive fusariosis is a devastating disease, with high mortality rate, especially when the disease is disseminated, with various metastatic skin lesions and lung involvement [21]. Although the outcome is largely influenced by immune status of the host [22], much of its poor prognosis may be related to the fact that fungal burden is very high when the diagnosis is established. With this regard, the use of a serum biomarker that allows clinicians to early start antifungal treatment may have a potential benefit. This is what has been observed with invasive aspergillosis [4], [23], [24]. In the present study we observed that GMI was positive before the diagnosis of invasive fusariosis in 73% of the 15 cases in which GMI was positive, at a median of 10 days, and even before the first clinical manifestation attributed to fusariosis in 53%. Furthermore, seven of these 11 patients had at least two positive GMI tests before the diagnosis (≥4 tests in 5 patients). These data suggest that if an antifungal agent active against *Fusarium* spp. is started as a strategy of pre-emptive (or diagnostic-driven) approach based on positive GMI tests [25], the disease can be treated earlier, with a potential to improve the outcome.

In the present study the fastest way of reaching the diagnosis of invasive fusariosis was by direct exam of biological materials (especially skin biopsy), with the finding of hyaline hyphae obtained just a few hours after the diagnostic procedure. Clinicians should be very aggressive in promptly performing biopsy in the presence of suspicious skin lesions, and appropriately handle the biologic material, sending part of the biopsy for direct exam and culture, and part for histopathology.

The kinetics of GMI after treatment has been increasingly reported as an important tool to assess outcome, with better survival in patients who have rapid normalization of GMI [26]–[31]. In the present study we could not establish any correlation between the kinetics of GMI and the outcome, with 37.5% of patients who had normalization of GMI surviving 90 days after the diagnosis of invasive fusariosis, compared with 29% of those who did not normalize GMI. Reasons for not observing any correlation may be due to the strong influence of host factors on the outcome of invasive fusariosis [22], and the small number of patients analyzed.

Our study has important implications for clinical practice. Positive GMI tests in high-risk patients should be interpreted as indicative of invasive aspergillosis and invasive fusariosis, especially in centers/regions in which invasive fusariosis is more prevalent. Likewise, a strategy of initiation of an antifungal agent based on positive GMI and its ability to abort the overt clinical manifestations of invasive fusariosis should be explored.

In conclusion, we showed that the majority of patients with invasive fusariosis have positive GMI tests, and the test becomes positive before the diagnosis of fusariosis in most cases.
